# Patterns of use of oral antivirals for COVID-19 in people 70 years and over in Victoria, Australia: a linked data study

**DOI:** 10.1186/s12879-026-12536-y

**Published:** 2026-01-23

**Authors:** Laura J. Edwards, Anish Scaria, Allen C. Cheng, Janaki Amin, Elizabeth J. Robinson, Caroline Sumpton, Bette Liu

**Affiliations:** 1 School of Population Health, UNSW Sydney, Sydney, Australia; 2 National Centre for Immunisation Research and Surveillance, Sydney, Australia; 3 Centre for Epidemiology and Evidence, NSW Health, Sydney, Australia; 4 Infectious Diseases, Monash Health, Melbourne, Australia; 5 School of Clinical Sciences, Monash University, Melbourne, Australia; 6 Department of Health Sciences, Macquarie University, Sydney, Australia; 7 Health Protection NSW, NSW Health, Sydney, Australia; 8 Department of Health, Victoria, Australia; 9 Faculty of Medicine and Health, University of Sydney, Sydney, Australia

**Keywords:** COVID-19, SARS-CoV-2, Nirmatrelvir-ritonavir, Molnupiravir, Antiviral agents

## Abstract

**Background:**

Two oral antivirals for treatment of COVID-19 are available in Australia, nirmatrelvir-ritonavir, which is recommended, and molnupiravir, which is approved for use when nirmatrelvir-ritonavir is contraindicated. From 2022 to 2024, over 1.5 million Pharmaceutical Benefits Scheme (PBS) prescriptions were dispensed. We examined dispensing of antivirals in people aged 70 + years in Victoria, Australia, from July 2022 to April 2023.

**Methods:**

We linked COVID-19 notifications in residents of Victoria (population 6.6 million) aged 70 + years to data from the Australian 2021 Census of Population and Housing, Australian Immunisation Register, PBS and Medicare Benefits Scheme. We examined trends over time and compared the socio-demographic and clinical characteristics of people who received antivirals with those who received no treatment, according to the type of antiviral received. We also estimated the proportion of people that received molnupiravir and nirmatrelvir-ritonavir who had a pharmacological contraindication to nirmatrelvir-ritonavir.

**Results:**

Among 76,120 people aged 70 + years with a COVID-19 notification, 50,005 (66%) received an antiviral with 37,101 (74%) receiving molnupiravir. The proportion of people that received nirmatrelvir-ritonavir increased during the study period from 19% (1957/10,256) in July 2022 to 35% (720/2075) in April 2023. After adjusting for age and sex, characteristics associated with receipt of antivirals included RACF residence, higher income and higher education. Compared to molnupiravir, receipt of nirmatrelvir-ritonavir was greater in those with higher incomes, higher education and no COVID-19 vaccine booster in the last 6 months. The majority of people who received molnupiravir (25,378/37101;68%) did not have a category 1 pharmacological contraindication to nirmatrelvir-ritonavir.

**Conclusions:**

During the study period, most COVID-19 cases in Victoria received antivirals but variations according to age, sex, socioeconomic status, residence in aged care and vaccination suggest that further efforts to identify barriers and enablers to treatment are warranted. Our findings also suggest that pharmacological contraindications to nirmatrelvir-ritonavir are not the primary reason for prescribing molnupiravir.

**Supplementary Information:**

The online version contains supplementary material available at 10.1186/s12879-026-12536-y.

## Introduction

Since its emergence in 2019, SARS-CoV-2 has been one of the leading contributors to the global burden of disease, causing 5000 deaths in Australia in 2023 [1, 2]. Treatment of COVID-19, along with vaccination, are crucial strategies to reduce the ongoing impact of the virus. Two oral antivirals for COVID-19, molnupiravir (Lagevrio) and nirmatrelvir-ritonavir (Paxlovid) are currently available. First approved for use by the Therapeutic Goods Administration (TGA) in January 2022, and subsequently listed on the Pharmaceutical Benefits Scheme (PBS) in March (molnupiravir) and May (nirmatrelvir-ritonavir) 2022, they rapidly became two of the most prescribed drugs in Australia. In 2022-23 and 2023-24 there were 891,000 and 633,000 prescriptions dispensed respectively. At approximately $1000 per course for each drug, the cost to the Australian Government from mid-2022 to mid-2024 was $1.75 billion [3]. Medicines on the PBS are subsidised for those who meet the eligibility criteria (see below) with a co-payment ranging from approximately $7 (for concession card holders) to $30 (for non concession card holders).

People who test positive for COVID-19 at high risk for severe outcomes are eligible for oral antivirals under a PBS authority prescription if commenced within 5 days of symptom onset [4]. Since 11 July 2022, this eligible population has included all adults aged 70 + years; people who are moderately to severely immunocompromised; and adults aged 50 + years or Aboriginal and Torres Strait Islanders aged 30 + years with risk factors for severe disease (such as chronic heart disease, chronic respiratory conditions and diabetes) [4]. 

The initial PBS eligibility criteria were for either nirmatrelvir-ritonavir or molnupiravir with no preferred treatment. Since late 2022, following publication of the PANORAMIC trial [5], which found that molnupiravir was not effective at reducing severe COVID-19 outcomes in vaccinated patients, nirmatrelvir-ritonavir has been recommended as the first line treatment for COVID-19 [6]. In January 2023, the PBS eligibility criteria changed to restrict molnupiravir use to situations when nirmatrelvir-ritonavir was contraindicated [7]. People not enrolled with Medicare or who don’t meet the PBS eligibility criteria can access antivirals with a private prescription at a cost of over $1000 [8].

Several studies have identified that prescribing of antivirals by clinicians often differs from clinical recommendations, such as prescribing outside of recommended timeframes, repeat prescribing and use in individuals with contraindications [9–14]. Additionally, variations in the supply of antivirals by age, sex, socio-economic indicators, and cultural and linguistic diversity have been reported, with females, older people, and people from more advantaged socio-economic areas more likely to receive treatment [9, 12, 15–19]. Studies to date have largely described differences in treatment according to the type of antiviral but have not compared treatment with no treatment in eligible groups and little is known about antiviral usage patterns in people over 70 years who were notified cases of COVID-19. We aimed to describe patterns of PBS dispensing of molnupiravir, nirmatrelvir-ritonavir, or no treatment in relation to notified cases of COVID-19 in Victoria from July 2022 to April 2023.

## Methods

### Setting and data sources

We conducted a population-based observational study using routinely collected data for individuals in Victoria, Australia, aged 70 years and older between 11 July 2022 and 30 April 2023. Victoria is Australia’s second most populous jurisdiction with an estimated population of 6.61 million in June 2022 [20]. We chose the study start date to align with the period when antivirals became available on the PBS for all adults 70 years and over and the end date based on data availability.

We used the Person-Level Integrated Data Asset (PLIDA) managed by the Australian Bureau of Statistics (ABS), which combines information on health, education, government payments, income and taxation, employment and population demographics, including the 2021 Census of Population and Housing (the Census). Datasets are linked securely using a unique person-linkage spine by the ABS and then de-identified data are accessible for analysis by researchers. For this study, we used linked data from the Victorian Department of Health COVID-19 surveillance system (Transmission and Response Epidemiology Victoria, TREVi), the Census, Australian Immunisation Register (AIR), Pharmaceutical Benefits Scheme (PBS), Medicare Benefits Scheme (MBS), and Residential Aged Care Facility (RACF) data.

TREVi surveillance data includes all notified COVID-19 cases in Victoria, along with demographic and clinical information. In Victoria, pathology services are required to notify the department of all laboratory-confirmed cases [21]. Until October 2022, positive Rapid Antigen Tests (RATs) were required to be notified through an online self-completed registration process. After that period, results could be voluntarily notified to the Department of Health.

The Census is conducted by the ABS. Data used in this study included age, sex, educational attainment, weekly income, area of residence, country of birth and language spoken at home.

Medicare is the Australian Government scheme that funds access to healthcare and medications for people who are living in Australia. Australian citizens, permanent residents and people from selected countries with reciprocal agreements with Australia are eligible [22]. 

The AIR is a national register of vaccines administered in Australia to people enrolled with Medicare that contains information on the brand, dose and date of vaccination. The number and timing of COVID-19 vaccines (< 6 months or ≥ 6 months) were used in this analysis.

The PBS, funded by the Australian Government, provides subsidised medicines to Australian residents enrolled with Medicare and some overseas visitors. The PBS Schedule is the list of medicines available in Australia to eligible people at a government-subsidised price. All medicines dispensed under the Scheme are recorded including the item, formulation, dose and date of dispensing. PBS data were used to determine the type of COVID-19 antiviral used, dispensing date, and to identify dispensing of drugs contraindicated with nirmatrelvir-ritonavir in the 90 days prior to COVID-19 diagnosis. We were only able to identify pharmacological contraindications to the use of nirmatrelvir-ritonavir using PBS data as we did not have data on clinical contraindications (e.g. severe kidney impairment and severe liver disease).

The Rx-risk Comorbidity Index (Rx-risk) is a validated measure of comorbidities that is calculated based on an individual’s pharmaceutical history [23]. We also used PBS data to calculate Rx-risk as has been done previously [24]. The MBS records all health services funded by Medicare. Information on the number of general practice visits in the previous 12 months was extracted. Aged care data included information on the individuals residing in a RACF. More detailed information on these datasets and linkage are available in an earlier publication [24].

### Study population

For our main analysis we included notified cases of COVID-19 in Victoria with a calculated onset date between 11 July 2022 and 30 April 2023 who were aged 70 + years at 11 July 2022 and resident in Victoria. The commencement date of 11 July 2022 was chosen to coincide with the expansion of PBS eligibility criteria for oral antivirals to include all people in Australia aged 70 + years regardless of comorbidity or vaccination status. For the secondary analysis, we included all individuals in Victoria aged 70 + years who had a PBS dispensing record of molnupiravir or nirmatrelvir-ritonavir.

### Study definitions

The primary outcome of interest was treatment with either of the two oral antivirals available on the PBS for COVID-19, nirmatrelvir-ritonavir (PBS item code 12996B) and molnupiravir (PBS item code 12910 L). For the purposes of this study, the calculated date of onset of COVID-19 was defined as the earliest of the following dates: date of onset of symptoms, earliest positive specimen date (or specimen received date). Treatment was considered to have occurred if there was a record of dispensing within the 2 days prior to and up to 7 days after the calculated onset date of COVID-19. This dispensing window, while broader than the PBS criteria, was selected after examining how PBS antiviral dispensing dates compared to the COVID-19 calculated onset date. The onset date could be subject to inaccuracies because it is self-reported after the event. We only included the first dispensing of an antiviral for people with a COVID-19 notification in the study period; any subsequent dispensings were excluded.

The individual characteristics of interest included age (in years), sex (male, female, other/unknown), geographical remoteness (based on an ABS classification; major city, regional or remote area), highest level of education attained (Certificate or lower, Diploma or higher, no education/unknown), weekly household income (<$1000, ≥$1000, unknown), language spoken at home (English, other, unknown), country of birth (Australia; overseas non-English-speaking [including countries other than New Zealand, United Kingdom, Canada, Ireland, and United States], other [English-speaking], unknown), number of comorbidities calculated according to Rx-risk (0–1, 2–3, 4–5, ≥ 6), number of GP visits in the previous 12 months (0–2, 3–6, 7–12, > 12), COVID-19 vaccination status (< 3 doses, ≥ 3 doses and recent booster dose within 6 months, ≥ 3 doses and recent booster dose > 6 months ago) and nirmatrelvir-ritonavir pharmacological contraindications (category 1: do not co-administer, category 2: stop or replace drug if possible, category 3: potential interaction) using the Liverpool Drug Interaction Resource [25]. This approach has been used previously [9]. 

### Analysis

We first examined trends in monthly counts of COVID-19 notifications and PBS dispensing records of nirmatrelvir-ritonavir or molnupiravir. We examined both the proportion of the COVID-19 notifications with a PBS antiviral dispensing record within the − 2 days to + 7 days of the COVID-19 onset date as well as overall PBS antiviral dispensing. We calculated the proportion of people who received antivirals within 3 and 5 days of the onset date of COVID-19.

For our primary analysis, among those who had a COVID-19 notification, we compared the characteristics of those who received a PBS antiviral dispensation within − 2 and + 7 days of the COVID-19 calculated onset date with those who did not receive an antiviral dispensation and the type of antiviral received. We estimated age and sex adjusted risk ratios using log-binomial regression to compare the socio-demographic characteristics associated with (i) receiving an oral antiviral versus none and (ii) by type of antiviral (nirmatrelvir-ritonavir versus molnupiravir). For these analyses, we grouped categorical variables into two categories only (e.g. COVID-19 vaccine booster dose ≥ 6 months earlier or < 3 doses, compared with COVID-19 vaccine booster dose within the previous 6 months) and we excluded missing or unknown results to enable us to generate risk ratios adjusted for age and sex.

For our secondary analysis, we compared the characteristics of Victorian residents aged 70 + years who had a PBS record of antiviral dispensing but no temporally related COVID-19 notification record with those who did have a COVID-19 notification.

Analyses were conducted using SAS version 9.3 and *R* (version 4.4.1). The study received a Notification of Exemption from Human Research Ethics Review from the Research Ethics and Integrity Committee at the University of Queensland including a waiver of consent from participants. To maintain confidentiality, data were de-identified prior to being provided for this research in accordance with Australian privacy legislation [26]. 

## Results

### Study population

**Fig. 1:**
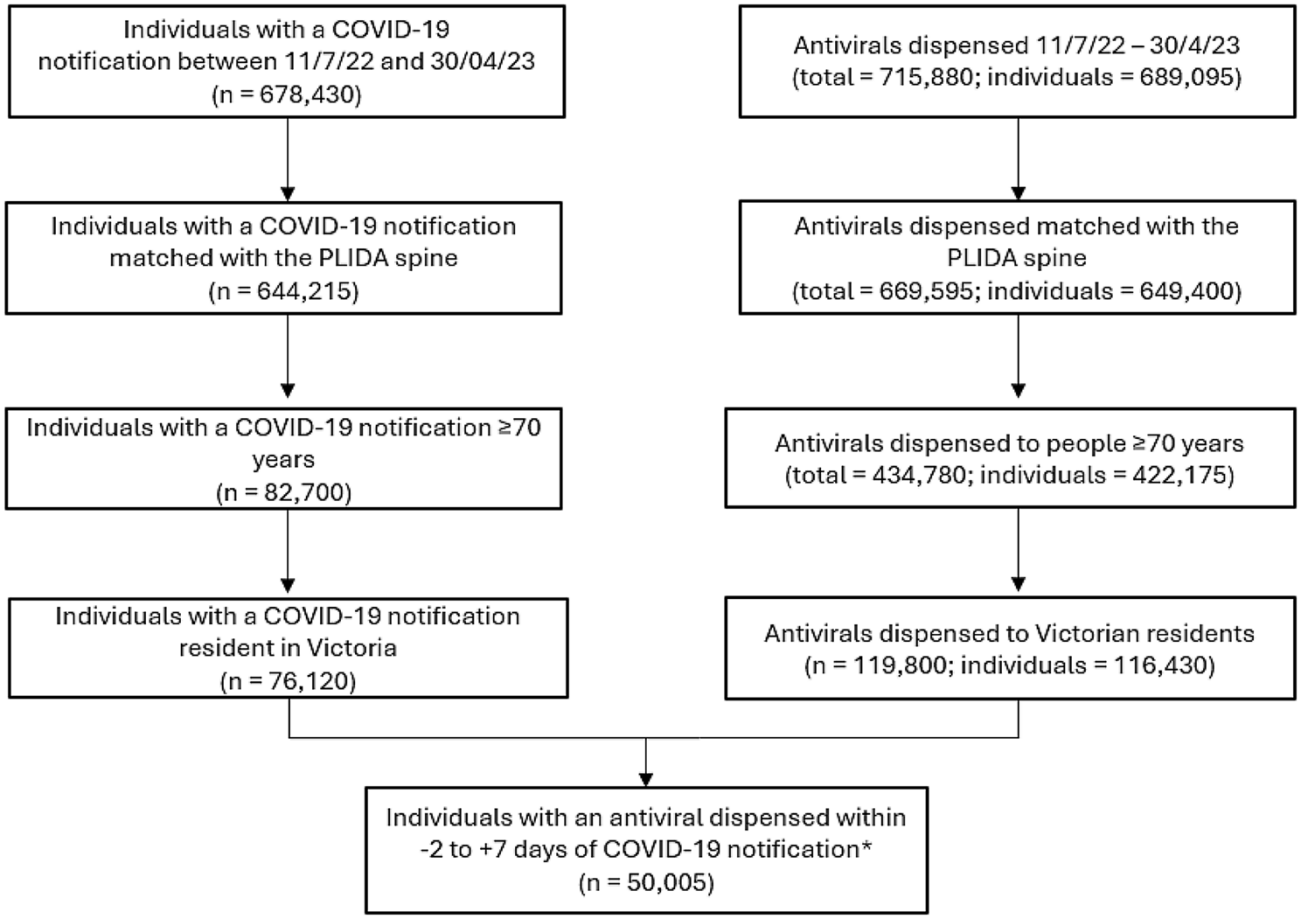
Flow chart describing the selection of the study population and linked records of antiviral dispensing. *Only antivirals dispensed between 11 July 2022 and 30 April 2023 were included therefore people at the start and end of the study period did not have the full range (-2 to + 7 days) of dispensings included

Figure 1 shows the selection of the study population from the different linked records. After removing non-Victorian residents and people aged under 70 years, there were 116,430 individuals with an antiviral dispensed. Of these, 50,005 individuals had an antiviral dispensed in the 2 days prior up to 7 days after their COVID-19 notification, and 66,425 individuals did not have a COVID-19 notification (*Only antivirals dispensed between 11 July 2022 and 30 April 2023 were included therefore people at the start and end of the study period did not have the full range (-2 to + 7 days) of dispensings included (*Only antivirals dispensed between 11 July 2022 and 30 April 2023 were included therefore people at the start and end of the study period did not have the full range (-2 to +7 days) of dispensings included. Figure 1).

### Changes in notifications and the supply of antivirals over the study period

**Fig. 2:**
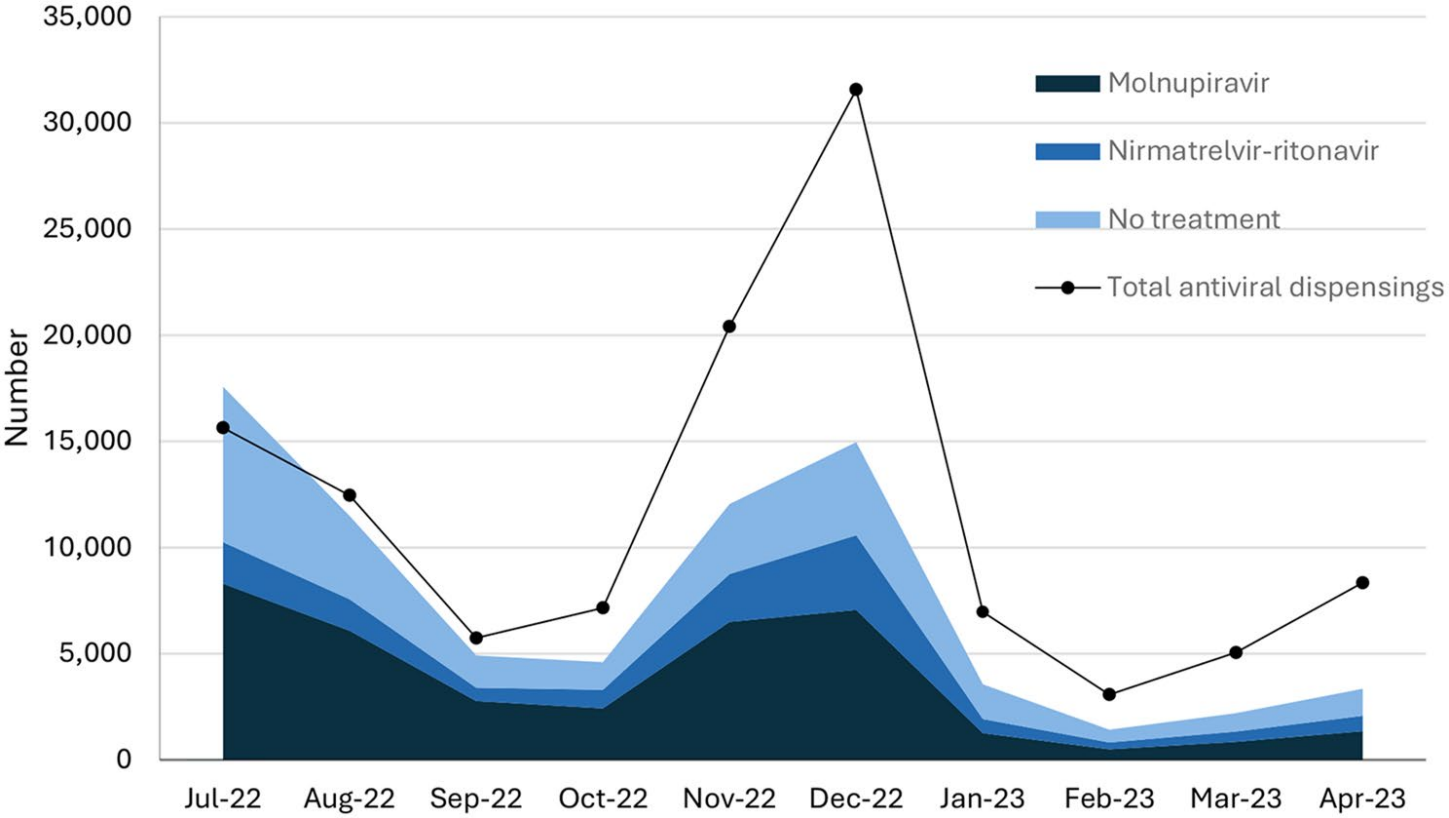
Number of cases of COVID-19 that received molnupiravir, nirmatrelvir-ritonavir or no treatment, and total antivirals. Shows the monthly counts of individuals with a COVID-19 notification, who received molnupiravir, nirmatrelvir-ritonavir or no treatment and count of all individuals with a COVID-19 oral antiviral dispensed according to PBS data, Victorian residents aged 70 + years, July 2022 to April 2023

The number of people with a COVID-19 notification each month during the study period ranged from 17,576 in July 2022 to 1417 in February 2023. The percentage of people with a COVID-19 notification that received an antiviral increased from 58% (10,256/17,576) in July 2022 to 73% (8749/12,047) in November 2022 before decreasing again to 54% (1927/3561) in January 2023. The proportion of notified cases who received an antiviral and were supplied with nirmatrelvir-ritonavir increased over the study period from 19% (1957/10,256) in July 2022 to 35% (720/2075) in April 2023 (Figure 2).

The total number of antivirals dispensed broadly followed the number of COVID-19 notifications but there was an increase in dispensings relative to notifications over the study period. July 2022 was the only month with fewer antiviral dispensings than notifications (15,643 vs. 17,576)(Figure 2). From July to September 2022, when positive RAT registration was mandatory, the proportion of antivirals dispensed without a COVID-19 notification was around 37% (12,622/33,842). From October 2022, when positive RAT registration became voluntary, the proportion of antivirals dispensed without a COVID-19 notification exceeded antivirals dispensed with a notification at 65% (53,801/ 82,585)(Fig. 2).

### Timing of treatment

**Fig. 3:**
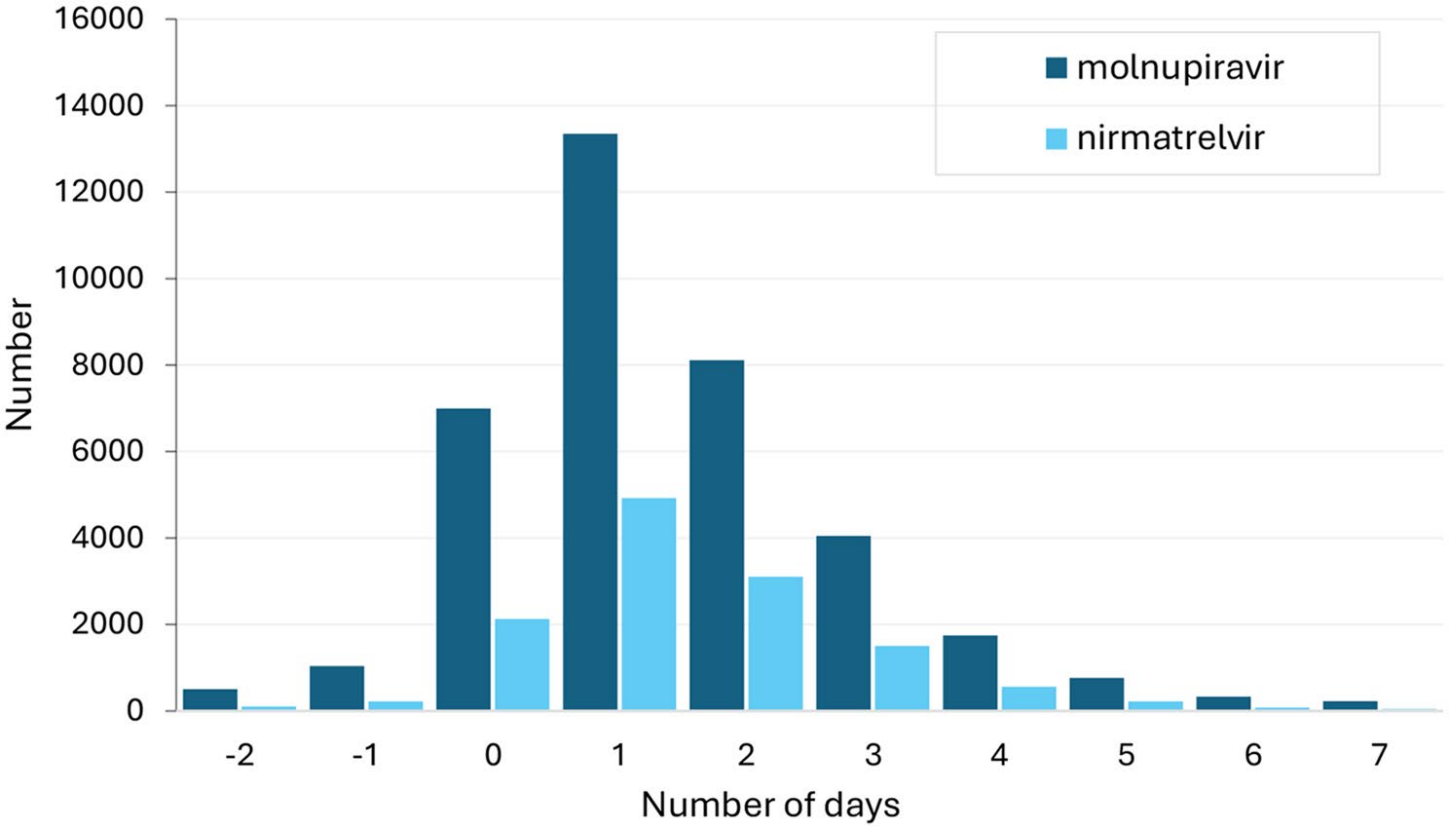
Number of days between onset of COVID-19 and dispensing of molnupiravir and nirmatrelvir-ritonavir. Shows the number of prescriptions for molnupiravir and nirmatrelvir-ritonavir each day in the 2 days before, day of, and 7 days after the onset date of COVID-19 notification, Victorian residents aged 70 + years, July 2022 – April 2023

The majority of people with a COVID-19 notification who received antivirals had them dispensed within 3 days (91.7% of molnupiravir recipients; 92.9% nirmatrelvir-ritonavir recipients) and almost all received them within 5 days (98.6% of molnupiravir recipients; 99.0% of nirmatrelvir-ritonavir recipients) (Fig. 3).

### Characteristics of those with COVID-19 who received antiviral treatment

Of the 76,120 people with a COVID-19 notification over the study period, 50,005 (65.7%) had a temporally associated PBS-supplied antiviral of whom 37,107 (74.2%) received molnupiravir, and 12,897 (25.8%) received nirmatrelvir-ritonavir. Table 1: Characteristics of cases of COVID-19 according to receipt of antivirals and type of antiviral.Table 1 shows the characteristics of the population according to receipt of antiviral. Of those who received antivirals, a higher proportion were female, had a higher income, had higher education, spoke English at home and were born in Australia. Receipt of an antiviral was also more likely in those with comorbidities, more frequent GP visits and a more recent COVID-19 vaccine booster.

Among those who received an antiviral, people who received nirmatrelvir-ritonavir were more likely to be female, have a higher income and higher level of education. They were more likely to speak English at home and be born in Australia. They were less likely to: be aged 80 + years, reside in a RACF, have had more than 12 GP visits in the previous year, have more comorbidities and have received a COVID-19 booster in the previous 6 months.

Of the 12,897 people who had dispensing records of nirmatrelvir-ritonavir, 2250 (17.4%) had been dispensed a category 1 drug contraindicated with nirmatrelvir-ritonavir in the previous 90 days. Of the 37,107 people who had dispensing records of molnupiravir, most 25,378 (68.4%) had not received a category 1 drug contraindicated with nirmatrelvir-ritonavir in the preceding 90 days (Table 1).

The characteristics of people who had an antiviral dispensed without a COVID-19 notification in Victoria differed from those with a COVID-19 notification according to vaccination status, income, education, comorbidities and residence in aged care. Those without a COVID-19 notification were more likely to have received < 3 COVID-19 vaccine doses at 11,430/43,059 (26.5%) of molnupiravir recipients and 4905/19,082 (25.7%) of nirmatrelvir-ritonavir recipients compared to people with a COVID-19 notification (4.3% [1613/37,107] of molnupiravir recipients and 3.1% [402/12,897] of nirmatrelvir-ritonavir recipients). They were also less likely to have had a booster in the previous 6 months. Higher income, higher educational attainment, presence of comorbidities and residence in aged care were less common in people without a notification (supplementary Table 1).

**Table 1:** Characteristics of cases of COVID-19 according to receipt of antivirals and type of antiviral

	Any antiviral	No antiviral treatment	Molnupiravir	Nirmatrelvir-ritonavir
	*N* (% of category)	*N* (% of category)	*N* (% of category)	*N* (% of category)
**Overall**	50,005 (100)	26,115 (100)	37,107 (100)	12,897 (100)
**Demographics**				
**Age group**				
70–74	16,200 (32.4)	8,354 (32.0)	10,835 (29.2)	5,365 (41.6)
75–79	13,266 (26.5)	6,370 (24.4)	9,455 (25.5)	3,811 (29.5)
80–84	9,105 (18.2)	4,648 (17.8)	7,055 (19.0)	2,050 (15.9)
85+	11,432 (22.9)	6,743 (25.8)	9,761 (26.3)	1,671 (13.0)
**Female sex**	28,254 (56.5)	13,542 (51.9)	20,887 (56.3)	7,367 (57.1)
**RACF resident**				
No	42,223 (84.4)	22,384 (85.7)	29,773 (80.2)	12,450 (96.5)
Yes	7,781 (15.6)	3,731 (14.3)	7,337 (19.8)	444 (3.4)
**Geographical remoteness**				
Major Cities of Australia	36,272 (72.5)	19,197 (73.5)	26,940 (72.6)	9,332 (72.4)
Other areas	13,734 (27.5)	6,914 (26.5)	10,169 (27.4)	3,565 (27.6)
**Socioeconomic characteristics**				
**Household income (weekly)**				
< 1000	28,823 (57.6)	16,104 (61.7)	21,063 (56.8)	7,760 (60.2)
> 1000	11,626 (23.3)	4,947 (18.9)	7,796 (21.0)	3,830 (29.7)
Any Other	9,562 (19.1)	5,066 (19.4)	8,252 (22.2)	1,310 (10.2)
**Highest education level attained**				
Certificates and lower	27,450 (54.9)	16,031 (61.4)	21,096 (56.9)	6,354 (49.3)
Diploma or higher	15,522 (31.0)	5,882 (22.5)	10,292 (27.7)	5,230 (40.6)
No education or unknown	8,117 (16.2)	3,120 (11.9)	6,646 (17.9)	1,471 (11.4)
**Language spoken at home**				
English	40,796 (81.6)	19,144 (73.3)	29,736 (80.1)	11,060 (85.8)
Any other	9,175 (18.3)	6,935 (26.6)	7,345 (19.8)	1,830 (14.2)
Not Stated	36 (0.1)	38 (0.1)	29 (0.1)	7 (0.1)
**Country of birth**				
Australia	31,856 (63.7)	14,465 (55.4)	23,221 (62.6)	8,635 (67.0)
Other non-English speaking*	12,537 (25.1)	8,849 (33.9)	9,775 (26.3)	2,762 (21.4)
Other (English-speaking)	5,566 (11.1)	2,766 (10.6)	4,085 (11.0)	1,481 (11.5)
Not Stated	45 (0.1)	34 (0.1)	28 (0.1)	17 (0.1)
**Clinical characteristics**				
**Rx risk (co-morbidity score)**				
0–1	2,506 (5.0)	1,892 (7.2)	1,584 (4.3)	922 (7.1)
2–3	9,331 (18.7)	4,798 (18.4)	6,019 (16.2)	3,312 (25.7)
4–5	15,749 (31.5)	7,618 (29.2)	11,074 (29.8)	4,675 (36.2)
>=6	22,416 (44.8)	11,804 (45.2)	18,432 (49.7)	3,984 (30.9)
**Number of GP visits in year prior**				
0–2	2,506 (5.0)	1,892 (7.2)	1,584 (4.3)	922 (7.1)
3–6	9,331 (18.7)	4,798 (18.4)	6,019 (16.2)	3,312 (25.7)
7–12	15,749 (31.5)	7,618 (29.2)	11,074 (29.8)	4,675 (36.2)
>=13	22,416 (44.8)	11,804 (45.2)	18,432 (49.7)	3,984 (30.9)
**COVID-19 Vaccination**				
< 3 doses	2,015 (4.0)	2,918 (11.2)	1,613 (4.3)	402 (3.1)
Booster ≤ 6 months	35,664 (71.3)	16,765 (64.2)	27,167 (73.2)	8,497 (65.9)
Booster > 6 months	12,326 (24.7)	6,429 (24.6)	8,325 (22.4)	4,001 (31.0)
**Pharmacological contraindications to nirmatrelvir-ritonavir~**				
Any contraindication	36,472 (72.9)	18,899 (72.4)	28,736 (57.5)	7,736 (29.6)
Category 1	13,979 (28.0)	7,438 (28.5)	11,729 (31.6)	2,250 (17.4)
Category 2	27,697 (55.4)	14,509 (55.6)	21,932 (59.1)	5,765 (44.7)
Category 3	12,597 (25.2)	6,569 (25.2)	10,031 (27.0)	2,566 (19.9)
Nil	13,532 (27.1)	7,215 (27.6)	8,371 (22.6)	5,161 (40.0)

*English speaking countries included New Zealand, United Kingdom, Canada, Ireland and United States

#Categories of contraindicated drugs are not mutually exclusive

~One or more drugs contraindicated with nirmatrelvir-ritonavir dispensed in the previous 90 days

Legend: Table 1 Shows the number and proportion of notified cases of COVID-19 aged 70 years and older who received any antiviral, no treatment, nirmatrelvir-ritonavir or molnupiravir according to demographic, socioeconomic, clinical, vaccination and pharmacological characteristics, Victorian residents, July 2022 to April 2023

### Characteristics associated with antiviral dispending

**Fig. 4:**
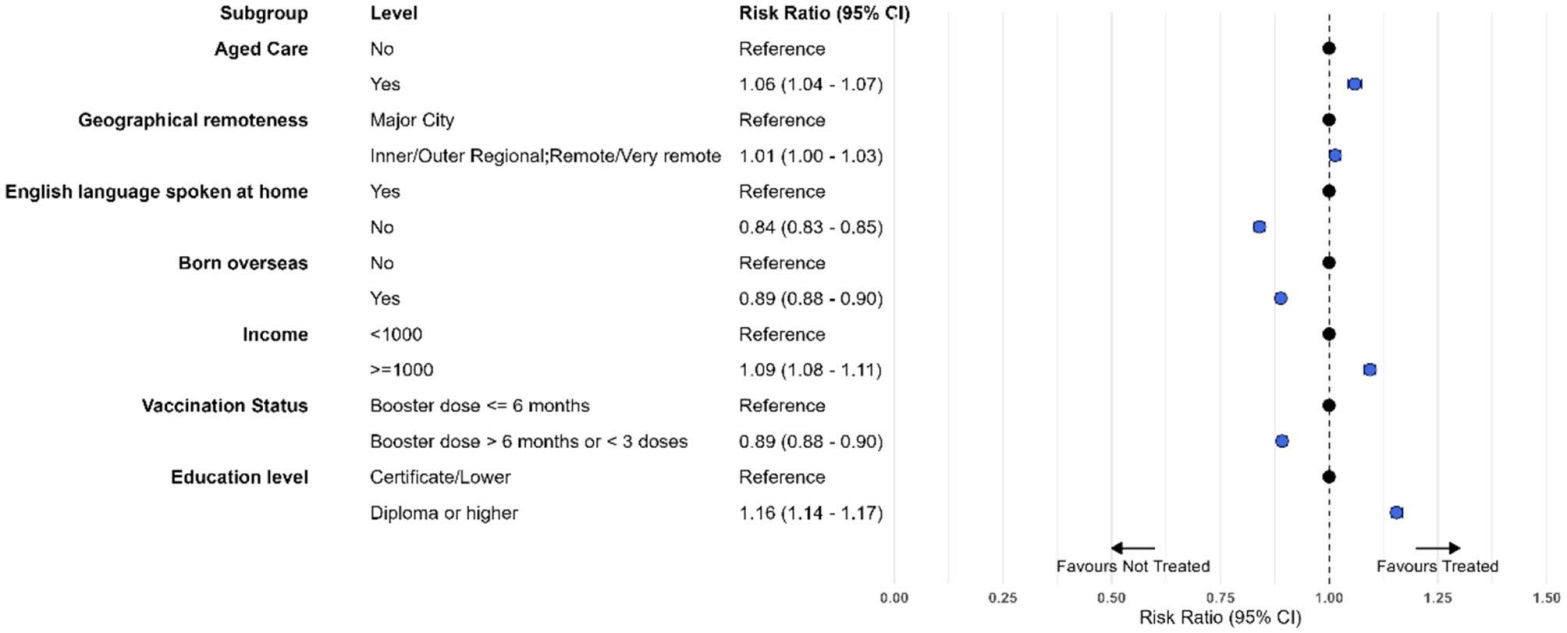
Characteristics associated with receiving oral antivirals compared with no treatment in Victorian resident cases aged 70 years and over, adjusted for age and sex, July 2022 to April 2023. Fig. 5 is a forest plot showing the risk ratio and 95% confidence interval for treatment with antivirals compared with no treatment for the characteristics of residence in aged care, geographical remoteness, English language spoken at home, being born oversease, weekly income, vaccination status and education level

After adjusting for age and sex, characteristics associated with greater likelihood of antiviral receipt included being resident in aged care (adjusted RR (aRR)1.06; 95% CI 1.04–1.07), higher income (aRR 1.09; 95% CI 1.08–1.11), and higher education (aRR 1.16; 95% CI 1.14–1.17). Antiviral receipt was less likely among people who spoke a language other than English at home (aRR 0.84; 95% CI 0.83–0.85), who were born outside of Australia (aRR 0.89; 95% CI 0.88–0.90) and who not had a COVID-19 vaccine in the last 6 months (aRR 0.89; 95% CI 0.88–0.90)(see Fig. 4).

**Fig. 5:**
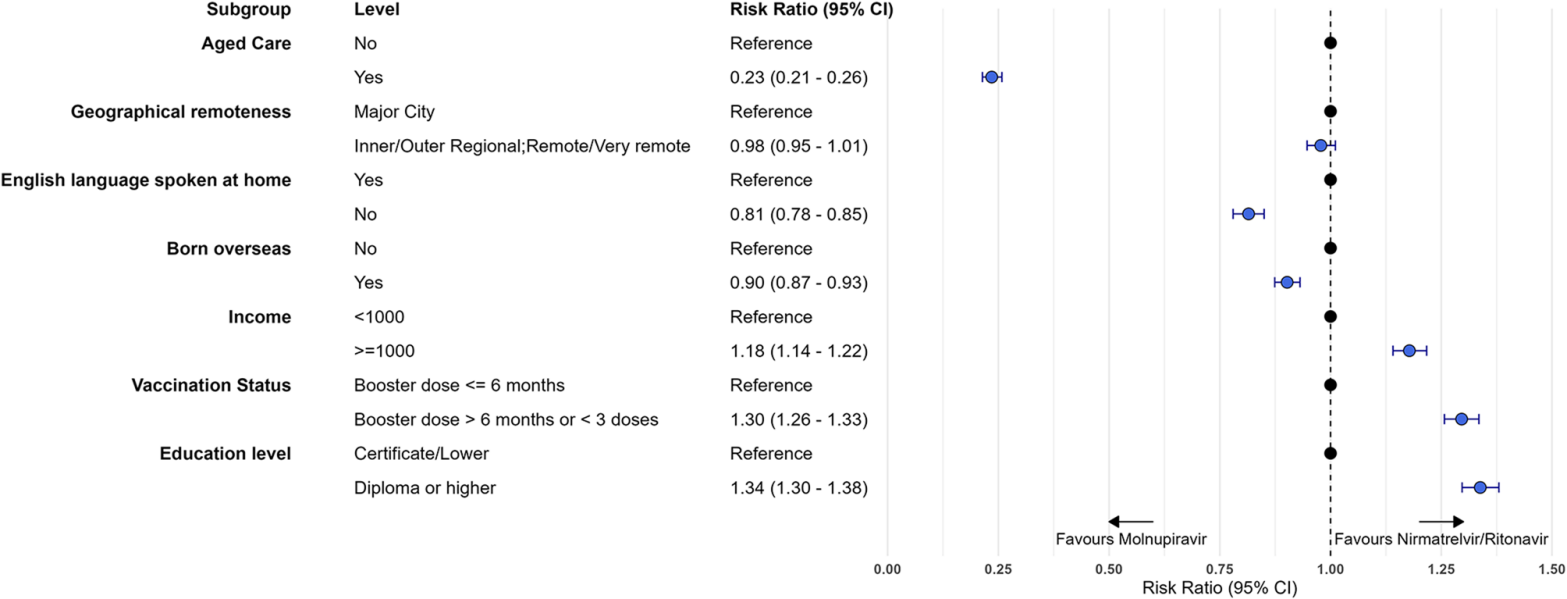
Characteristics associated with receiving nirmatrelvir-ritonavir compared with molnupiravir in Victorian resident cases aged 70 years and over, adjusted for age and sex, July 2022 to April 2023. Fig. 5 is a forest plot showing the risk ratio and 95% confidence interval for treatment with nirmatrelvir-ritonavir compared with molnupiravir for the characteristics of residence in aged care, geographical remoteness, English language spoken at home, being born oversease, weekly income, vaccination status and education level

Compared to those who received molnupiravir, receipt of nirmatrelvir-ritonavir was greater in those with higher incomes (aRR 1.18; 95% CI 1.14–1.22), higher education (aRR 1.34; 95% CI 1.30–1.38), no COVID-19 vaccine booster in last 6 months (aRR 1.30; 95% CI 1.26–1.33). It was lower in people who spoke a language other than English at home (aRR 0.81; 95% CI 0.78–0.85), in people born outside Australia (aRR 0.90; 95% CI 0.87–0.93) and substantially lower in those resident in an aged care facility (aRR 0.23; 95% CI 0.21–0.26)(Fig. 5).

## Discussion

In this study we found that almost two thirds of people aged 70 + years with a COVID-19 notification received oral antivirals between 2022 and early 2023 demonstrating that there is widespread acceptance by clinicians and eligible population groups. Molnupiravir was the most common treatment accounting for 74% of antiviral dispensings. Use of molnupiravir as a proportion of treatments dispensed decreased over time, in line with changes to recommendations regarding first line treatment, but by April 2023 it still accounted for 65% of antiviral dispensings. More females received antivirals than males (28,524 v’s 21,751). Just over two thirds of people who received molnupiravir (68%) did not have a category 1 pharmacological contraindication to nirmatrelvir-ritonavir.

After adjusting for both age and sex, we found differences in the socio-demographic and health characteristics of those who received antiviral compared with no treatment, and nirmatrelvir-ritonavir compared with molnupiravir. These differences were similar to common disparities observed in healthcare, with higher income, educational attainment, being born in Australia, and speaking English at home associated with greater likelihood of treatment and receipt of the first-line treatment (nirmatrelvir-ritonavir). Of interest, we did not find significant differences by geographical remoteness.

There were several novel findings in our study. First, people who had fewer than 3 doses or not received a COVID-19 vaccine booster in the previous six months were less likely to receive treatment (aRR 0.89; 95% CI 0.88–0.90). If they did receive treatment, it was more likely to be nirmatrelvir-ritonavir than molnupiravir (aRR 1.30; 95% CI 1.26–1.33). Second, RACF residents were more likely to receive treatment (aRR 1.06, 95% CI 1.04–1.07) but much less likely to receive nirmatrelvir-ritonavir than molnupiravir (aRR 0.23, 95% CI 0.21–0.26). It is plausible that the first novel finding is related to access to or uptake of acceptable healthcare services, as both vaccination and antivirals require access to healthcare practitioners. The reason for the adjusted risk ratio favouring nirmatrelvir-ritonavir among people who had not received a COVID-19 vaccine booster in the previous six months is unclear but could be related to other factors associated with likelihood of recent vaccination, such as comorbidities, as has been reported by other researchers [24].

Preferential molnupiravir use in RACF residents may be related to healthcare practitioner familiarity with molnupiravir, which was pre-positioned in RACF through the National Medical Stockpile by the Australian government prior to both nirmatrelvir-ritonavir and molnupiravir becoming available on the PBS; and also due to healthcare practitioner concerns regarding comorbidities or contraindications to the use of nirmatrelvir-ritonavir, which are more common among the elderly.

This is, to our knowledge, the first study in Australia comparing the characteristics of people aged 70 + years who were dispensed antiviral treatment with and without a COVID-19 notification. Overall, the number of people that received an antiviral with a COVID-19 notification was lower than the number of people that received an antiviral without a COVID-19 notification (50,004 vs. 62,141) demonstrating that case ascertainment was incomplete. The finding of an increase in the proportion of antivirals dispensed without a notification from October 2022 likely reflects the removal of the requirement to register RATs in Victoria from that time. We have limited data on the percentage of people aged 70 + in Victoria with COVID-19 symptoms that undergo lab testing. However, one study of test-seeking behaviour in Australia between 2021 and 2023 found that the probability of undergoing a test for COVID-19 was 37 to 73 per cent depending on the symptoms reported and the overall probability of reporting a positive RAT from 2022 to 2023 was 80 per cent [27].

Our findings indicate that the sociodemographic and clinical characteristics of notified cases of COVID-19 that received antivirals are broadly similar to all people aged 70 + years that received antiviral treatment with the exception of a marked difference in vaccination status. Those without a notification had fewer and less recent vaccinations. We also observed smaller differences in education, income and residence in aged care across the groups. Several other studies have found variations in prescribing and dispensing of antivirals by socioeconomic status [15–29]. Access to culturally-appropriate and affordable primary health care services is considered fundamental to reducing disparities in healthcare [16, 29]. The introduction of Medicare-funded telehealth at the start of the pandemic in Australia is reported to have supported access to timely treatment [30]. Addressing gaps in knowledge surrounding the use of antivirals, for both clinicians and consumers, has also been recommended by clinicians and researchers [15, 31].

An Australian study comparing treatment with no treatment among notified cases of COVID-19 found positive associations with female sex, older age, presence of comorbidities, COVID-19 vaccinations, and residing in a RACF [28]. International studies have found that use of molnupiravir rather than nirmatrelvir-ritonavir is associated with female sex, older age, presence of comorbidities, ethnicity, lower vaccination rates and residing in a RACF [9, 12, 32–34]. Our study, focussing on only those with a COVID-19 notification had mostly comparable results with earlier reports.

The finding of very small differences in the use of antivirals by geographical remoteness contrasts with other studies [15, 35]. Our analysis was adjusted for age and sex, which may explain the difference.

Several other studies have found that molnupiravir is the preferred antiviral and used more commonly than nirmatrelvir-ritonavir [9, 16, 28, 35]. This is despite estimates that only 22% of the population have a pharmacological or clinical contraindication to nirmatrelvir-ritonavir [9]. Our finding that 68% of people dispensed molnupiravir did not have a category 1 pharmacological contraindication to nirmatrelvir-ritonavir suggests that further efforts to understand the reasons for prescribing molnupiravir are needed. Avoidance of nirmatrelvir-ritonavir in situations where it wasn’t contraindicated has been demonstrated [13]. Factors reported to influence the use of nirmatrelvir-ritonavir include clinician knowledge, patient volumes and attitudes, availability, patient attitudes, and other clinical, social and demographic factors [11, 12]. Our finding that RACF residents were much less likely to receive nirmatrelvir-ritonavir than molnupiravir warrants further investigation.

It is promising that the proportion of people treated with antivirals that received nirmatrelvir-ritonavir increased over the study period from 19% to 35%. This has also been identified in other studies [9, 19]. We also found that 17% of patients dispensed nirmatrelvir-ritonavir had a category 1 contraindication suggesting that some patients may be receiving nirmatrelvir-ritonavir inappropriately. This is lower than the 28% reported by Lopez et al. but their cohort comprised all age groups and they included drugs dispensed in the 2 years prior to the antiviral, which likely explains the difference [9].

Our study strengths included the use of all COVID-19 notifications and PBS dispensing data in an area with a population of over 6 million people. The linked datasets enabled us to examine a large number of people and compare patterns of use over time and characteristics associated with antiviral use. We were able to analyse the characteristics of people with a COVID-19 notification who did not receive antivirals, which has not been done previously. This can be used to determine which groups may benefit from more targeted approaches to provision of antivirals in the future. We were also able to compare the characteristics of people who received antivirals with and without a COVID-19 case notification, which enabled us to gain a more comprehensive understanding of the representativeness of COVID-19 notifications and the patterns of antiviral use in Victoria. This also partially addressed the limitation of under-ascertainment of cases of COVID-19 that is inherent in surveillance data. However, one group that we did not have information on is people with COVID-19 that did not seek testing, that did not register a positive RAT, and that did not access antivirals. Understanding the characteristics of this group would be useful to determine who is eligible but missing out on COVID-19 antivirals.

There were several other limitations to this study. Firstly, we only included records of antivirals and other medications provided under the PBS. In February 2022, antivirals were supplied directly to RACFs across Australia under the National Medical Stockpile [36] and these may have been used after July 2022 in Victoria potentially resulting in underreporting of dispensing of antivirals in RACF in our study. We also did not have access to privately purchased antivirals although it is likely they represented a very small proportion of antivirals used due to the high cost.

We used the calculated onset date captured in TREVi which was a derived estimate using information on symptom onset where available, or otherwise reporting or testing data, and therefore may not represent the true date of symptom onset of COVID-19. It is likely that the actual time from symptom onset to commencement of antivirals was longer than what was captured in the calculated onset date, and hence our estimates of the timing from symptom onset to dispensing data may be inaccurate. Additionally, the date of dispensing may differ from the date of prescribing, and we were unable to assess whether drugs were taken as directed. However, other studies have found very high completion rates for antivirals [31].

We were only able to investigate pharmacological contraindications to nirmatrelvir-ritonavir and we did not have information on the clinical contraindications (severe chronic kidney disease and severe liver disease) which would have enabled us to determine more accurately whether the PBS eligibility criteria were followed. We were also unable to determine if clinicians arranged to withhold these drugs, which is an approach that can be utilised for some of the category 1 drugs. Some drugs, such as St John’s Wort, are not available under the PBS, therefore we may have missed some people who had contraindications to nirmatrelvir-ritonavir.

Our study was conducted in Victoria and may not be representative of the rest of Australia with some studies suggesting higher antiviral prescribing and use of nirmatrelvir-ritonavir in Victoria than NSW [9, 19]. Our study was also limited to notification data current to April 2023—it is unclear if our findings would translate to oral antiviral use in 2025. Additionally, COVID-19 notifications underestimated infections so our findings among notified cases may not be representative of the broader population. However, our findings were similar across all PBS dispensings—for both notified and non-notified cases—suggesting they are generalisable.

Our finding that 34 per cent of people aged 70 + years with a COVID-19 notification did not receive a temporally associated antiviral suggests that gaps remain in access and/or supply within Australia. It may also relate to the acceptability or affordability of antivirals, with most people in Australia having to make a co-payment for prescriptions medicines. Reported barriers to receiving antivirals include being unable to access a healthcare practitioner, testing late in the illness, or not knowing that antivirals were an option [31]. Reported reasons for clinicians not prescribing antivirals include late presentation, patients declining treatment, or asymptomatic/mild disease [37]. Strategies to address these barriers and to increase the use of nirmatrelvir-ritonavir, such as streamlining the process to assess for contraindicated medications through integrated decision support tools and clinician education, may enhance the appropriate use of antivirals in Australia.

## Conclusion

Oral antivirals for COVID-19 were widely used in Victorians aged 70 years and over from mid-2022 to early 2023. We found small but significant differences in demographic and socioeconomic characteristics among notified cases of COVID-19 in both the dispensing of any antiviral compared to no treatment and in dispensing of nirmatrelvir-ritonavir compared with molnupiravir. Further efforts to understand the reasons for these differences, and to address how utilisation could be improved and made more equitable are warranted. Continuing to monitor antiviral use in Australia through repeated analysis of linked data can provide a more comprehensive picture of antiviral use and document changes in practice over time. This will ensure treatment is reaching the groups for whom it is intended and that harms from the virus are reduced.

## Supplementary Information

Below is the link to the electronic supplementary material.

Supplementary Material 1

## Data Availability

The study investigators do not own the data. Requests for use of de-identified data should be directed to the Australian Bureau of Statistics.
