# Supplementary material for: Patterns of use of oral antivirals for COVID-19 in people 70 years and over in Victoria, Australia: a linked data study
Source: BMC Infect Dis. 2026 Jan 23;26:408. doi: 10.1186/s12879-026-12536-y (PMC12918484; doi:10.1186/s12879-026-12536-y)
Supplement: Supplementary file 1 — Supplementary Material 1 [file 12879_2026_12536_MOESM1_ESM.docx]

# Supplementary data

**Table 1: Characteristics of Victorian residents 70 years and over who received antivirals with and without a COVID-19 notification, July 2022 to April 2023**

|  | **Molnupiravir without C19 notification** |  | **Nirmatrelvir-ritonavir without C19 notification** |  | **Molnupiravir with C19 notification** |  | **Nirmatrelvir-ritonavir with C19 notification** |  |
| --- | --- | --- | --- | --- | --- | --- | --- | --- |
|  | N | % of category | N | % of category | N | % of category | N | % of category |
| **Overall** | 43,059 | 100 | 19082 | 100 | 37,107 | 100 | 12,897 | 100 |
| **Median age** |  |  |  |  |  |  |  |  |
| 70-74 | 13,750 | 31.9 | 7,515 | 39.4 | 10,835 | 29.2 | 5,365 | 41.6 |
| 75-79 | 12,708 | 29.5 | 5,890 | 30.9 | 9,455 | 25.5 | 3,811 | 29.5 |
| 80-84 | 8,872 | 20.6 | 3,364 | 17.6 | 7,055 | 19.0 | 2,050 | 15.9 |
| 85+ | 7,730 | 18.0 | 2,315 | 12.1 | 9,761 | 26.3 | 1,671 | 13.0 |
| **Female sex** | 23,839 | 55.4 | 11,039 | 57.9 | 20,887 | 56.3 | 7,367 | 57.1 |
| **Resident in aged care** |  |  |  |  |  |  |  |  |
| No | 41,161 | 95.6 | 18,935 | 99.2 | 29,773 | 80.2 | 12,450 | 96.5 |
| Yes | 1,896 | 4.4 | 146 | 0.8 | 7,337 | 19.8 | 444 | 3.4 |
| **Geographical remoteness** |  |  |  |  |  |  |  |  |
| Major Cities of Australia | 29,028 | 67.4 | 13,364 | 70.0 | 26,940 | 72.6 | 9,332 | 72.4 |
| Other areas | 14,037 | 32.6 | 5,718 | 30.0 | 10,169 | 27.4 | 3,565 | 27.6 |
| **Household income (weekly)** |  |  |  |  |  |  |  |  |
| <1000 | 28,620 | 66.5 | 12,030 | 63.0 | 21,063 | 56.8 | 7,760 | 60.2 |
| >1000 | 9,126 | 21.2 | 5,235 | 27.4 | 7,796 | 21.0 | 3,830 | 29.7 |
| Any Other | 5,317 | 12.3 | 1,820 | 9.5 | 8,252 | 22.2 | 1,310 | 10.2 |
| **Highest education level attained** |  |  |  |  |  |  |  |  |
| Certificates and lower | 26,974 | 62.6 | 10,802 | 56.6 | 21,096 | 56.9 | 6,354 | 49.3 |
| Diploma or higher | 10,374 | 24.1 | 6,182 | 32.4 | 10,292 | 27.7 | 5,230 | 40.6 |
| No education or unknown | 5,713 | 13.3 | 2,097 | 11.0 | 6,646 | 17.9 | 1,471 | 11.4 |
| **Language spoken at home** |  |  |  |  |  |  |  |  |
| English | 33,219 | 77.1 | 15,460 | 81.0 | 29,736 | 80.1 | 11,060 | 85.8 |
| Any other | 9,804 | 22.8 | 3,611 | 18.9 | 7,345 | 19.8 | 1,830 | 14.2 |
| Not Stated | 33 | 0.1 | 9 | 0.0 | 29 | 0.1 | 7 | 0.1 |
| **Country of birth** |  |  |  |  |  |  |  |  |
| Australia | 25,520 | 59.3 | 11,904 | 62.4 | 23,221 | 62.6 | 8,635 | 67.0 |
| Overseas non-English speaking* | 13,145 | 30.5 | 5,197 | 27.2 | 9,775 | 26.3 | 2,762 | 21.4 |
| Any Other | 4,321 | 10.0 | 1,959 | 10.3 | 4,085 | 11.0 | 1,481 | 11.5 |
| **Rx risk (co-morbidity score)** |  |  |  |  |  |  |  |  |
| 0-1 | 3,671 | 8.5 | 3,309 | 17.3 | 1,584 | 4.3 | 922 | 7.1 |
| 2-3 | 10,910 | 25.3 | 6,276 | 32.9 | 6,019 | 16.2 | 3,312 | 25.7 |
| 4-5 | 13,329 | 31.0 | 5,347 | 28.0 | 11,074 | 29.8 | 4,675 | 36.2 |
| ≥6 | 15,158 | 35.2 | 4,151 | 21.8 | 18,432 | 49.7 | 3,984 | 30.9 |
| **Number of GP visits in year prior** |  |  |  |  |  |  |  |  |
| 0-2 | 1,849 | 4.3 | 1,293 | 6.8 | 1,584 | 4.3 | 922 | 7.1 |
| 3-6 | 7,810 | 18.1 | 4,947 | 25.9 | 6,019 | 16.2 | 3,312 | 25.7 |
| 7-12 | 14,736 | 34.2 | 6,926 | 36.3 | 11,074 | 29.8 | 4,675 | 36.2 |
| >12 | 18,668 | 43.4 | 5,916 | 31.0 | 18,432 | 49.7 | 3,984 | 30.9 |
| **COVID-19 Vaccination** |  |  |  |  |  |  |  |  |
| <3 doses | 11,430 | 26.5 | 4,905 | 25.7 | 1,613 | 4.3 | 402 | 3.1 |
| Booster <6 months | 2,822 | 6.6 | 1,110 | 5.8 | 27,167 | 73.2 | 8,497 | 65.9 |
| Booster ≥6 months | 28,805 | 66.9 | 13,064 | 68.5 | 8,325 | 22.4 | 4,001 | 31.0 |
| **Prescription in last 90 days for drug contraindicated with nirmatrelvir-ritonavir** |  |  |  |  |  |  |  |  |
| Any contraindication | 33,962 | 78.9 | 11,991 | 62.8 | 28,736 | 57.5 | 7,736 | 29.6 |
| Category 1 | 13,731 | 31.9 | 3,565 | 18.7 | 11,729 | 31.6 | 2,250 | 17.4 |
| Category 2 | 25,874 | 60.1 | 8,947 | 46.9 | 21,932 | 59.1 | 5,765 | 44.7 |
| Category 3 | 12,762 | 29.6 | 4,084 | 21.4 | 10,031 | 27.0 | 2,566 | 19.9 |
| Nil | 9,097 | 21.1 | 7,091 | 37.2 | 8,371 | 22.6 | 5,161 | 40.0 |

Legend: This table shows the characteristics of Victorian residents 70 years and over who received molnupiravir or nirmatrelvir-ritonavir with and without a COVID-19 notification from July 2022 to April 2023.
